# Determination of the Forming Limit for a ZIRLO™ Sheet with High Anisotropy

**DOI:** 10.3390/ma13245743

**Published:** 2020-12-16

**Authors:** Minsoo Kim, Seokmoo Hong

**Affiliations:** Department of Mechanical and Automotive Engineering, Kongju National University, Cheonan 31080, Korea; mkim@kongju.ac.kr

**Keywords:** zirconium alloy, forming limit, anisotropy, yield criterion, triaxiality failure diagram

## Abstract

In this study, the experimental two-dimensional forming limit diagram (FLD) data for a ZIRLO™ sheet, which is used in nuclear fuel rod support grids, were converted and presented as a triaxiality failure diagram (TFD). Most previous studies assumed ZIRLO™ to be isotropic when calculating the effective stress and strain. However, for highly anisotropic materials, the anisotropy should be considered for calculations of effective stress and strain; hence, in this study, they were calculated by introducing the normal anisotropy coefficient. To obtain this parameter of the ZIRLO™ specimens, tensile tests were performed on specimens with 0°, 45°, and 90° angles with respect to the rolling direction. It was observed that the average normal anisotropy coefficient measured during the tests was 4.94, which is very high. The von Mises isotropic and Hill 48 anisotropic yield criterion were applied to the FLD data that were experimentally determined using a limit dome height test and were converted into effective stress and effective strain. When the FLD is converted to TFD, the curve will increase in the top-right direction if the *r*-value is greater than 1, and this become more severe as the *r*-value increases. The TFD, which was converted considering the anisotropy, is almost the same to the TFD obtained using the digital image correlation method in the tensile tests of four specimens with different stress states. If anisotropy is not considered, then the formability is normally underestimated. However, a highly accurate TFD can be obtained with the method proposed in this study.

## 1. Introduction

The forming limit represents the ability of a metal to transform into the desired shape without necking or breaking. The forming limit diagram (FLD) is a diagram that exhibits the limit strains for the strains from uniaxial tension to equibiaxial tension based on an assumed linear strain path in terms of the minor strain *ε*_2_ (*x*-axis) and the major strain *ε*_1_ (*y*-axis) [1,2]. Then, the limit strain values can be connected by a single line to obtain the forming limit curve (FLC). The FLD is widely used to predict the formability of sheet metals owing to its simple concept and ease of use. In particular, the FLD is experimentally determined through the limit dome height test (LDHT), in which the specimen is deformed by a hemispherical punch till it fractures. A single hemispherical punch is raised or lowered to stretch the square-shaped sheet specimen. At this time, beads are used to completely fix the specimen, and a dog-bone type specimen is sometimes used instead of a square specimen so that fracture occurs in the center of the specimen. During this test, different deformation paths can be easily implemented by varying the width of the specimen. The strain of the specimen can be measured by the deformation of a square or circular grid printed on the specimen surface. According to ISO 12004 guidelines [3], a Gaussian regression (inverse quadratic curve) of the strains measured from a fractured specimen is performed, and the peak of the curve is regarded as the fracture limit. Because this peak value represents just the estimated strain between the local necking and ductile fracture, it cannot represent the actual behavior of the material [4]. Additionally, as the friction between the specimen and the dome mold can directly affect the deformations, an exact forming limit cannot be obtained. Previous studies have reported several drawbacks of the LDHT. Because of the friction caused by the contact between the punch and the blank, experiments need to be repeated to obtain consistent results, and above all, the friction can cause material changes during the experiment [5,6]. Moreover, the punch pressure can act as a contact condition that overestimates the material formability [7,8]. Thus, the process of experimentally measuring the forming limit is complicated, which requires excessive time and cost. As a result, studies have been actively conducted to measure the theoretical forming limit, such as the necking criteria proposed by Hill [9] and Swift [10], the Marciniak–Kuczynski model [11], and the modified maximum force proposed by Hora et al. [12]. However, as the theoretical equations are complex and are linked to complicated finite element analysis, metal forming technicians may not actually be able to use them.

**Figure 1:**
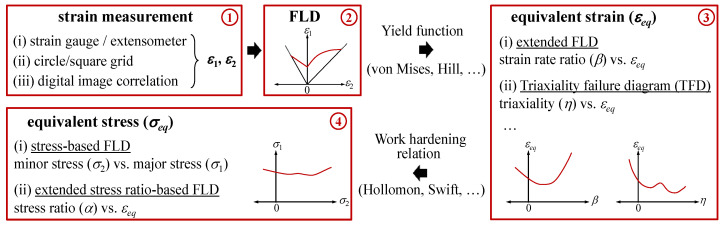
Schematic diagram of the procedure to determine path-independent forming limit criteria.

Generally, during forming, sheet metals are deformed along a nonlinear path. However, because the FLC assumes a path dependence, path-independent forming limit criteria are required in multistage deep-drawing forming or hydroforming processes for most of the sheet materials. As such, various forming limit criteria have been developed to overcome the dependence of the FLD on the strain path. Figure 1 shows a schematic diagram of the process of developing path-independent forming limit criteria applicable for in sheet metal forming. While stress measurement is very challenging, the strain can be measured easily using strain gauges, extensometers, and grid markings. With the development of digital image correlation (DIC) technology, displacement and strain can be more accurately measured through image analyses based on time increments. Forming limits can be obtained using the experimentally measured strains in the major direction (*ε*_1_) and minor direction (*ε*_2_) for various strain patterns. In addition, by applying the appropriate yield criterion and hardening law to the obtained strain value, it can be converted into effective strain (*ε_eq_*), effective stress (*σ_eq_*), triaxiality (*η*), and other parameters and can be expressed in terms of various path-independent forming limits. During a tensile test, diffusion necking begins at the maximum load point, but the forming limit should be obtained based on local necking. Therefore, the material model for sheet metal forming should account for the stress–strain behavior of the material, including the uniform elongation, local necking, and fracture. Previous studies have shown that various empirical stress–strain relations such as the Swift and Voce models can effectively extrapolate the actual stress–strain curve up to failure, which has been verified by a bulge test [13]. However, Kim et al. [14] showed that the stress–strain relationship of ZIRLO™ materials can be expressed using an empirical stress–strain relationship until diffusion necking but cannot be expressed by extrapolation after that point. Therefore, when the path-independent strain/stress-based forming limit criterion is calculated to avoid the strain path dependence of the FLD, inherent calculation errors can arise owing to the selection of the yield criterion and hardening law. Paul [15] showed that these calculation errors in the strain-based forming limit criterion are caused by the yield criterion, whereas in the stress-based criterion, they are caused by the hardening law as well as the yield criterion. When various forming limit criteria are converted based on the experimental FLD, the effect of material anisotropy is almost excluded. However, when anisotropy is extremely high, such as in case of as ZIRLO™ materials, it must be considered while calculating the forming limit.

In this study, a method for converting the experimentally obtained FLD to a triaxiality failure diagram (TFD) is proposed. First, ZIRLO™ sheets are prepared in three different directions with respect to the rolling direction to be used as specimens, and they are subjected to a tensile test for obtaining the anisotropy coefficient. The procedure and results for obtaining the experimental FLD are described in Section 2 along with the method of obtaining the anisotropy coefficient. In Section 3, von Mises and Hill 48 [16] yield criteria are applied to the (*ε*_1_, *ε*_2_) pair of FLD, and the process of converting them to *ε_eq_*, strain ratio (*β* ≡ *ε*_2_/*ε*_1_), and *η* is described in detail. Based on the *ε_eq_* and *η* values calculated from the aforementioned two yield criteria, it is shown that the two-dimensional FLD data converted to TFD, which is a three-dimensional fracture limit, are significantly different owing to the effect of anisotropy. In Section 4, the TFD is obtained for four specimens with different properties, and the results are compared to verify the accuracy of the theoretically converted TFD considering anisotropy. Finally, conclusions are presented in Section 5.

## 2. Experimental Setup

### 2.1. Tensile Tests

**Figure 2:**
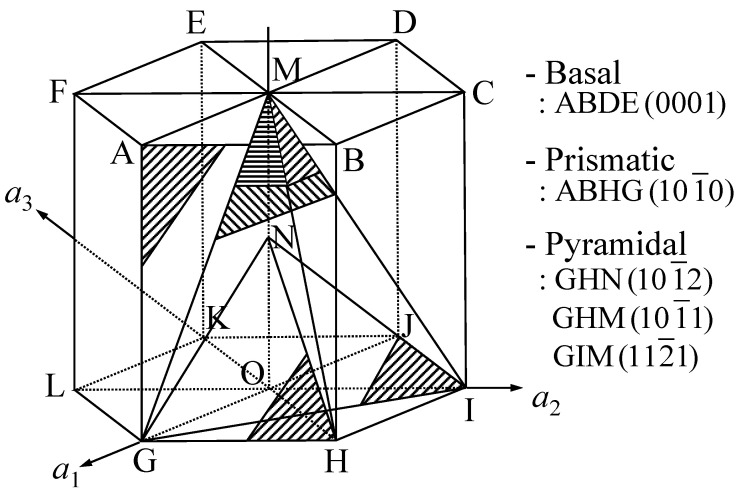
Important planes in a hexagonal close-packed (HCP) structure.

Zirconium alloys have excellent mechanical strength and corrosion resistance as well as low neutron absorption cross-section, so they are used for fuel element cladding and spacer grid in light water reactors to improve the stability of the fuel assembly structure [17]. One of the zirconium alloys, ZIRLO™ with Zr-1.0Sn-1.0Nb-0.1Fe (wt%) chemical composition, was developed by Westinghouse Company (Cranberry Township, PA, USA) to improve growth and creep while further enhancing corrosion resistance. ZIRLO™ is a hexagonal close-packed (HCP) material with a limited number of slip systems. The main slip planes of the HCP structure are the base (0001), prism, and pyramid, and the corresponding slip directions and slip planes are shown in Figure 2. The basal plane (0001) in an HCP structure is the only plane with a high atomic density, and the diagonal axis $\left(10\overset{¯}{2}0\right)$ represents a high-density direction. In materials having HCP structures, such as zinc and magnesium, slip occurs in the (0001) plane along the $\left(10\overset{¯}{2}0\right)$ direction [18]. Because there is only one base surface per unit cell and three orientations, HCP materials contain three slip systems. Compared to the face-centered cubic (FCC) and body-centered cubic (BCC) materials, which have 12 and 48 slip systems, respectively, HCP materials have limited slip systems, and the twin crystals are significantly affected by the crystal orientation. As a result, a large anisotropy exists in the material.

**Figure 3:**
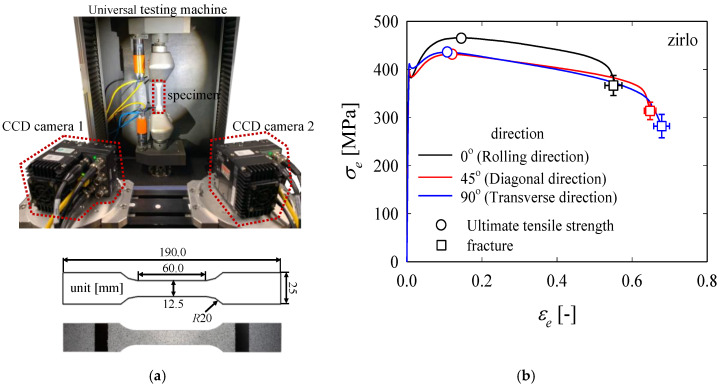
(**a**) Experimental setup and tensile test specimen. (**b**) Engineering stress vs. engineering strain curves with respect to the rolling direction (0°, 45°, and 90°).

The methods for measuring the anisotropy of a metal sheet is defined in ISO 10113 [19] and ASTM E517 [20]. If *ε_l_*, *ε_w_*, and *ε_t_* are the strains in the length, width, and thickness directions, respectively, the anisotropy coefficient *r* is defined by Equation (1). In general, because *ε_t_* is not measurable, it is calculated using Equation (2).

(1) $$r=\frac{\varepsilon_{w}}{\varepsilon_{t}}$$

(2) $$\varepsilon_{w}=\ln\left(\frac{w_{f}}{w_{0}}\right),\;\varepsilon_{t}=\ln\left(\frac{t_{f}}{t_{0}}\right)=\ln\left(\frac{l_{0}w_{0}}{l_{f}w_{f}}\right)\;\to \;r=\frac{\ln\left(w_{f}/w_{0}\right)}{\ln\left(l_{0}w_{0}/l_{f}w_{f}\right)},$$

where *l*, *w*, and *t* represent the gauge length of the longitudinal extensometer, gauge length of the transverse extensometer, and initial thickness of the material, respectively. The subscripts 0 and *f* indicate the time instances before and at the end of the test, respectively. In a previous study [4], it was reported that a square grid of 2 × 2 mm^2^ area and a 0.9 mm circular grid were printed on a tensile specimen to measure the strain, and the anisotropy was evaluated by calculating the degree of deformation of the grids remaining in the specimen before necking occurred. However, considering the environment where the material is used, it is difficult to apply the electrochemical etching method in grid printing due to its strong corrosion resistance, and moreover, the precision of the silk-screen printing method is very low. Above all, when the lattice deformation is evaluated, the subjectivity of the evaluator can affect the evaluation method, or the evaluation criteria can be quite different for different evaluation methods. As a result, the accuracy of the measured anisotropy coefficient can be low. When the 3D-DIC method is applied *ε_l_*, *ε_w_*, and *ε_t_* values in the region of interest can be obtained. Therefore, in this study, the DIC method was applied to obtain the strain in each direction for the entire specimen area using the ARAMIS 2019 [21] program, based on which the anisotropy coefficient was calculated. Tensile tests were performed 5 times for each specimen using the KS B 0801 [22] specimen on the universal testing machine 5ton Press Tester (Tomorrow Automotive Intelligent Electronics Core Technology Center, Cheonan, Korea) with a constant speed of 3.0 mm/min at room temperature. Two digital high-speed (900 frames/s) CCD cameras (Vision Research, Wayne, MI, USA) with a resolution of 1280 × 1024 pixels were used to measure the 3-D deformation of the ZIRLO sheet. The detailed information regarding the specimens, such as the shape and dimensions, is shown in Figure 3a, while the specimens with speckle patterns used for DIC application and the average nominal stress-nominal strain curves obtained during the five tensile tests are shown in Figure 3b. It is found that the uniform elongations of the rolling direction (RD), diagonal direction (DD), and transverse direction (TD) specimens were 14.6%, 12.1%, and 10.8%, respectively, whereas the maximum tensile strengths of the RD, DD, and TD specimens were 464.3, 431.0, and 435.6 MPa, respectively. For the anisotropy coefficients *r*_0_, *r*_45_, and *r*_90_, obtained from specimens with an angle of 0° (rolling direction, RD), 45° (diagonal direction, DD), and 90° (transverse direction, TD), respectively, with the rolling direction, the average normal anisotropy coefficient, $\bar{r}$, can be obtained using Equation (3), which is the anisotropy coefficient of the material in this study.

(3) $$\bar{r}=\frac{r_{0}+2r_{45}+r_{90}}{4}.$$

**Figure 4:**
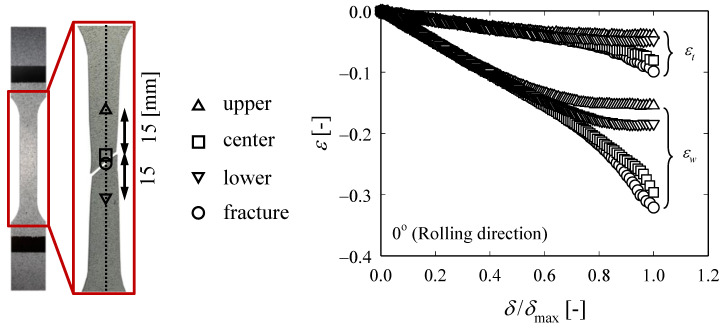
Evolution of *ε_w_* and *ε_t_* for various location from the uniaxial tensile tests at rolling direction (RD) specimen.

**Figure 5:**
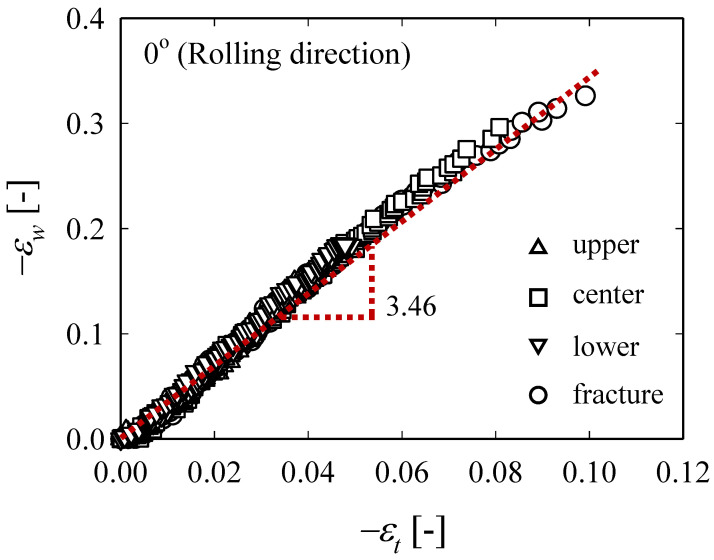
Comparison of the ratio of *ε_w_* and *ε_t_* for various location (RD specimen).

**Figure 6:**
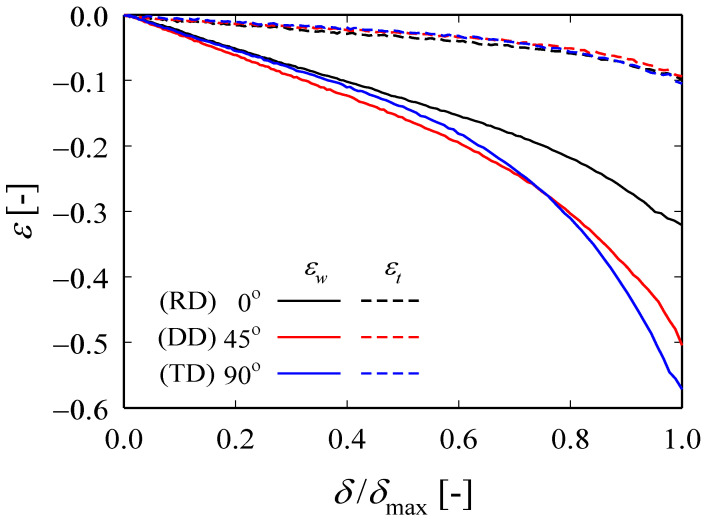
Evolution of *ε_w_* and *ε_t_* for fracture point from the uniaxial tensile tests at RD, diagonal direction (DD), and transverse direction (TD) specimen.

Figure 4 shows how *ε_w_* and *ε_t_*, which were measured at different points until the RD specimen breaks, evolves over strain, where *δ* represents the tensile test displacement and *δ*_max_ represents the displacement at the break. The measurement locations are the center of the specimen (□), a point 15 mm above and below the center of the specimen (△,▽), and the actual fracture point (○). In the ideal tensile test, the center of the specimen and the fracture point should coincide, but in the actual test, there are cases where they do not coincide due to various reasons. As shown in Figure 4, the evolution of *ε_w_* and *ε_t_* at each point proceeds identically, but the absolute size changes as the local necking begins, while the ratio (*ε_w_*/*ε_t_* ≡ *r*) is maintained the same (Figure 5). The negative representation of the legend in Figure 5 means that the width and thickness of the specimen decreases during the tensile test. Figure 6 shows the evolution of *ε_w_* and *ε_t_* obtained from the fracture points of RD, DD, and TD specimens. Regardless of the angle with respect to the rolling direction, *ε_t_* values of all specimens increase similarly, while *ε_w_* value of the TD specimen is the largest and that of the RD is the smallest (TD > DD > RD). The average values of *r*_0_, *r*_45_, and *r*_90_ were obtained in the five tests for each specimen and they are 3.46, 5.28, and 5.74, respectively. Furthermore, $\bar{r}$ was calculated using Equation (3) and it was 4.94, which indicates that ZIRLO™ is a material with very high anisotropy compared to other metallic materials.

### 2.2. Limit Dome Height Test

**Figure 7:**
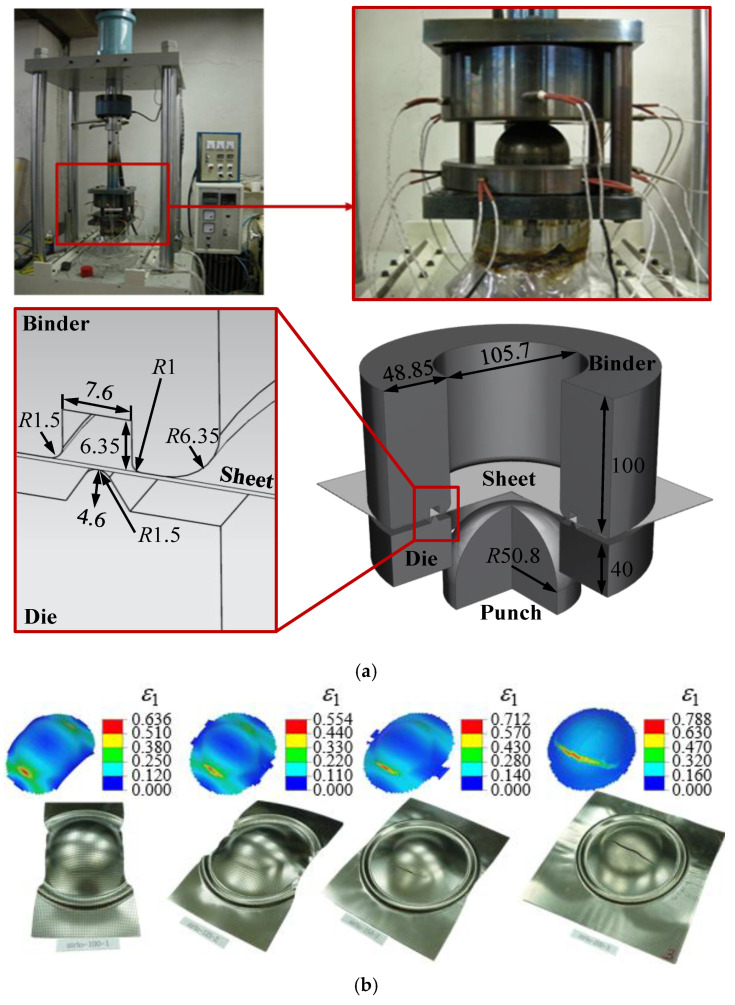
(**a**) Limit dome height (LDH) test machine and draw bead geometry. (**b**) Distributions of *ε*_1_ for 100, 125, 150, and 200-width specimens.

**Figure 8:**
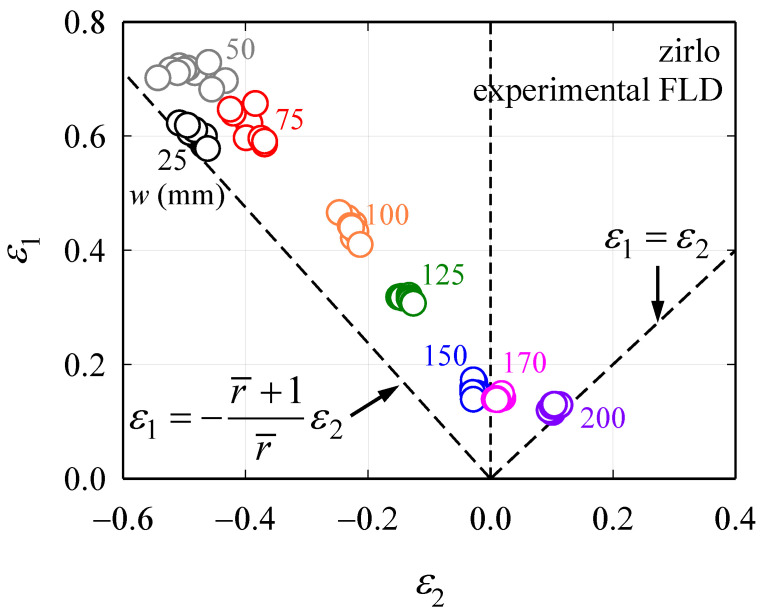
Forming limit diagram (FLD) for ZIRLO™.

To obtain the FLD of ZIRLO™, a limit dome height (LDH) test was performed with a hemispherical punch that had a diameter of 101.6 mm, as proposed by NUMISHEET 96 [23]. For the RD specimen having a thickness (*t*) = 0.48 mm and length (*l*) = 200 mm, eight rectangular and square specimens of same thickness and length but with different widths (*w*), which were 25, 50, 75, 100, 125, 150, 170, and 200 mm, were prepared. Equibiaxial tensile deformation occurs when the square specimen experiences negligible friction with the punch, and to realize this, a polyethylene resin was attached after tallow was applied to the punch and the contact part of the 200 mm-wide specimens. In the LDH test, a 300 kN binder force and a quasi-static punching speed (*v*) = 0.1 mm/s were used. As the punch rises, the center of the specimen deforms convexly, and the 0.9 mm circular grid was printed on the specimen surface by the silkscreen method for measuring the strain on it. Based on the amount of deformation of the grid printed on the surface, *ε*_1_ and *ε*_2_ values can be obtained for each specimen using the ARGUS [24] program as mentioned before. The *ε*_1_ and *ε*_2_ values are obtained for five sections in the center of the specimen, based on which the forming limit is calculated using the ISO 12004 [3] method, and detailed descriptions of this are explained in the study done by Kim et al. [25]. Figure 7 shows the test equipment, the shape of the specimen after the test, and their strain images obtained using ARGUS [24]. The FLD of each specimen was obtained from the 10 LDH tests. The results are shown in Figure 8. The limiting strain obtained for the 25 mm-wide specimen is placed on the simple tension line. When *w* ≥ 125 mm, the deviation of the limiting strain value is smaller than when *w* < 125 mm. This is because the alignment between the centers of the specimen and the punch during the test is relatively easy when *w* is high.

## 3. TFD with Anisotropy

Triaxiality (*η*) is defined as the ratio of the mean stress *σ_m_* to the effective stress *σ_eq_*, and it is used as a damage parameter in various damage models. *σ_m_* is expressed as

(4) $$\sigma_{m}\equiv \frac{1}{3}\left(\sigma_{1}+\sigma_{2}+\sigma_{3}\right).$$

Here, *σ*_1_, *σ*_2_, and *σ*_3_ are principal stresses in directions 1 (longitudinal), 2 (width), and 3 (thickness), respectively. Gurson [26] and Johnson and Cook [27] introduced a TFD where the horizontal and vertical axes represented *ε_eq_* and *η*, respectively, to characterize the fracture trajectories of materials under various stress conditions. They proposed a damage model in which *ε_eq_* monotonically decreases as *η* increases. However, their theory does not match the empirical results under compressive, shear, and biaxial tensile stresses. Therefore, Bao and Wierzbicki [28], Bai and Wierzbicki [29], and Lou and Huh [30] proposed modified ductile failure models and verified them through tests. Using different values of *η* and *ε_eq_* determined by the tensile test at the fracture point, it can be used as a prediction curve for failure in the tensile stress state. If the *ε_eq_* values are known for more *η* values, the accuracy of the fracture prediction curve increases.

Hill [16] proposed an anisotropic yield criterion where the effective stress can be calculated as in Equation (5). In 3D case, stress components are indicated with the indices 1, 2, and 3. *σ_ij_* defined as the stress in the *j* direction acting on a plane normal to the *i* direction with *i*, *j* = 1, 2, 3.

(5) $$\sigma_{eq}=\sqrt{\left[\frac{1}{2}\left\{F{\left(\sigma_{22}-\sigma_{33}\right)}^{2}+G{\left(\sigma_{33}-\sigma_{11}\right)}^{2}+H{\left(\sigma_{11}-\sigma_{22}\right)}^{2}\right\}+L\sigma_{23}^{2}+M\sigma_{31}^{2}+N\sigma_{12}^{2}\right]}.$$

Here, *F*, *G*, *H*, *L*, *M*, and *N* are six anisotropic parameters and can be expressed in terms of *r*_0_, *r*_45_, and *r*_90_, which were measured and calculated in the uniaxial tensile test. When *F*, *G,* and *H* values are equal to 1 and *L*, *M,* and *N* values equal to 3, the Hill 48 yield criterion becomes equal to the von Mises isotropic yield criterion. Generally, in the case of sheet metal forming, a plane stress condition is assumed, and Equation (5) is simplified to Equation (6) using $\bar{r}$

(6) $$\sigma_{eq}=\sqrt{\sigma_{1}^{2}+\sigma_{2}^{2}-\frac{2\bar{r}}{1+\bar{r}}\sigma_{1}\sigma_{2}}.$$

If *α* and *β* are defined as the stress ratio and strain ratio, respectively, the relationship between *α* and *β* can be expressed as Equation (7) [15]:

(7) $$\alpha \equiv \frac{\sigma_{2}}{\sigma_{1}}=\frac{\left(1+\bar{r}\right)\beta +\bar{r}}{\left(1+\bar{r}\right)+\bar{r}\beta },\;\beta \equiv \frac{\varepsilon_{2}}{\varepsilon_{1}}=\frac{\left(1+\bar{r}\right)\alpha -\bar{r}}{\left(1+\bar{r}\right)-\bar{r}\alpha }.$$

Equation (6) can be converted to Equation (8) using Equation (7), and thus *η* for the plane stress condition can be expressed as Equation (9).

(8) $$\sigma_{eq}=\sigma_{11}\sqrt{1+\alpha^{2}-\frac{2\bar{r}}{1+\bar{r}}\alpha }$$

(9) $$\eta \equiv \frac{\sigma_{m}}{\sigma_{eq}}=\frac{1+\frac{\left(1+\bar{r}\right)\beta +\bar{r}}{\left(1+\bar{r}\right)+\bar{r}\beta }}{3\sqrt{1+{\left(\frac{\left(1+\bar{r}\right)\beta +\bar{r}}{\left(1+\bar{r}\right)+\bar{r}\beta }\right)}^{2}-\left(\frac{2\bar{r}}{1+\bar{r}}\right)\left(\frac{\left(1+\bar{r}\right)\beta +\bar{r}}{\left(1+\bar{r}\right)+\bar{r}\beta }\right)}}.$$

Meanwhile, *ε_eq_* of the Hill 48 yield criterion can be rearranged using *ε*_1_, *β,* and $\bar{r}$ summarized in Equation (10).

(10) $$\varepsilon_{eq}=\varepsilon_{1}\frac{1+\bar{r}}{\sqrt{1+2\bar{r}}}\sqrt{1+\frac{2\bar{r}}{1+\bar{r}}\beta +\beta^{2}}.$$

**Figure 9:**
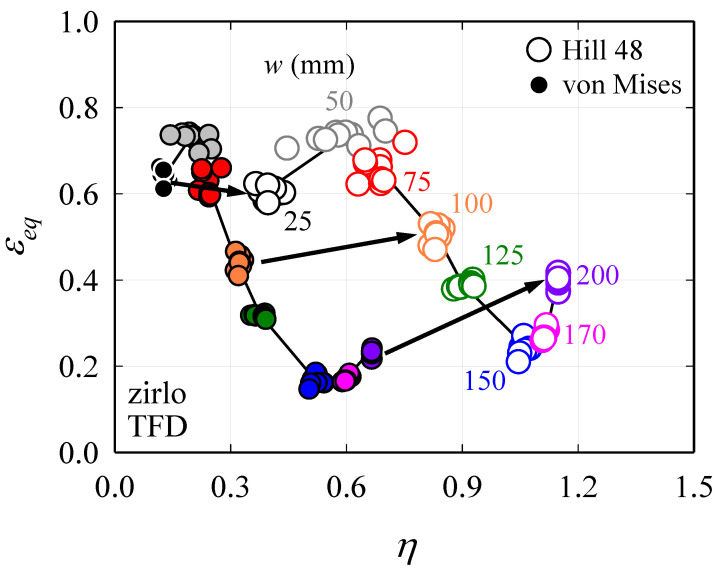
Influence of $\bar{r}$ when converting FLD data to triaxiality failure diagram (TFD).

Therefore, using Equations (9) and (10), the FLD can be converted to TFD using only the data obtained in Section 2.1 and Section 2.2 (i.e., *ε*_1_, *ε*_2_, and $\bar{r}$). Figure 9 shows the TFD converted from the FLD of ZIRLO™ shown in Figure 8, and it can be observed that the converted TFD varies significantly depending on the yield criterion. As indicated by the arrow given in Figure 9, the *η* value increases, and the TFD shifts to the right, which then increases as the width of the specimen increases. This is due to the consideration of the anisotropy. Additionally, *ε_eq_* also increases if the anisotropy is considered, except for the specimen with *w* = 25 mm, which indicates an improvement in the formability. As the stress–strain relationship of ZIRLO™ from necking to fracture does not follow the empirical equation proposed by Swift [10], Hollomon [31], and Voce [32], the error may be large when converting from the FLD-based forming limit stress curve [14]. Therefore, this study only deals with TFD conversion.

## 4. Validation through Tensile Tests

**Figure 10:**
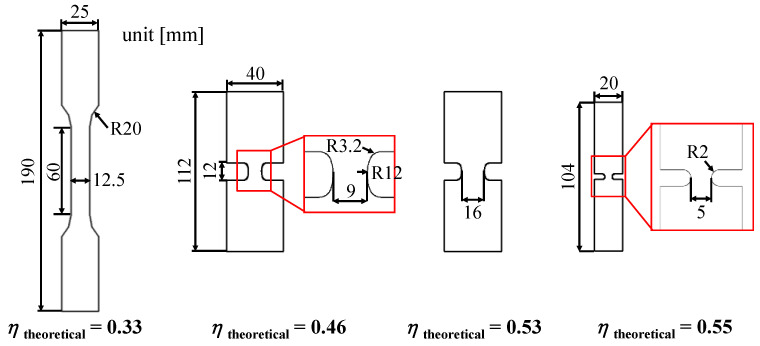
Specimen geometries for different stress state.

**Figure 11:**
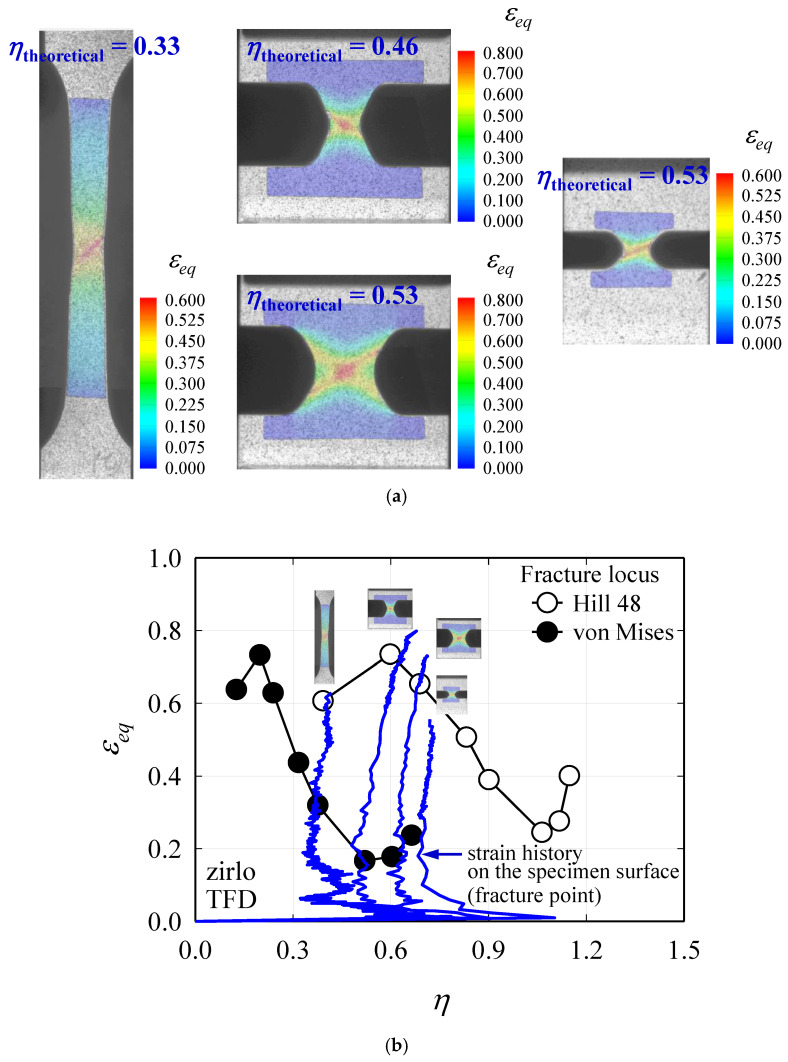
(**a**) Distribution of *ε_eq_* at fracture. (**b**) Comparison of forming limit with converted TFD.

To validate the accuracy of the TFD calculations shown in Section 3, tensile specimens capable of showing various stress states were prepared as shown in Figure 10, and the TFD curves of these specimens were obtained. The same 0.48 mm-RD plate made of ZIRLO™ was used as previously used to obtain the FLD. Specimens representing specific *η*_theoretical_ values were referenced from previous studies [33,34]. At this time, the *η*_theoretical_ values represented by each specimen are 0.33, 0.46, 0.53, and 0.55, respectively. The appropriate specimen shape for each stress state maintains a constant η value at the center of the specimen until fracture, which was verified by FEA or experiment. The DIC method was applied in the same manner where the anisotropy coefficient was obtained in the tensile test, and 1000 frames per second were measured using two CCD stereo cameras. The strain measurement was performed using the ARAMIS 2019 [21] program, and multiple images taken for the full field during the test allow accurate capturing of the fracture point as well as local strain measured at that point. Figure 11a shows the distribution of *ε_eq_* just before fracture of each specimen, whereas Figure 11b shows the *η* vs. *ε_eq_* curve obtained at the fracture point, which is overlapped in Figure 9. Comparing the TFD converted from FLD in consideration of anisotropy with the experimental fracture limit obtained from the specimen shown in Figure 10, it is almost the same as shown in Figure 11. When determining *η* in experimental studies related to TFD, $\bar{r}$ in Equation (9) is assumed to be 1. In the case of uniaxial tension in isotropic materials, theoretically *η* = 1/3 as *σ*_1_ = *σ_eq_* and *σ*_2_ = 0, while in the case of plane strain tension, *η* = 1/√3. Although specimens with *η* = 0.33, 0.46, 0.53, and 0.55 were used in the DP980 material, the *η* values of the corresponding ZIRLO™ specimens are 0.41, 0.65, 0.71, and 0.72, which are larger than those of DP980. This is because the known $\bar{r}$ value of DP980 is approximately 0.81–1 [35,36], which is much smaller than the $\bar{r}$ value of ZIRLO™, which is 4.94. Thus, it can be assumed to be almost relatively isotropic. If the anisotropy is not considered, the fracture limit is predicted to be too low for each stress state even if the friction generated in the LDH test for obtaining the FLD is considered. Therefore, in a highly anisotropic material such as ZIRLO™, the anisotropy must be considered for the TFD conversion.

## 5. Conclusions

The main purpose of this study was to measure the anisotropy of the ZIRLO™ specimen and convert the FLD to a TFD. Based on this study, the following conclusions can be drawn:

(1) The *r*_0_, *r*_45_, and *r*_90_ values of the ZIRLO™ specimens are 3.46, 5.28, and 5.74, respectively, which indicates that ZIRLO™ exhibits a high anisotropy compared to other metal materials. Moreover, its $\bar{r}$ value is 4.94. If DIC is used to calculate the $\bar{r}$ value, the ratio of the strain in the width direction and the strain in the thickness direction evolves to the same size until the specimen breaks even if the strains are measured at different points. As a result, a constant $\bar{r}$ value is obtained.

(2) The FLD obtained through the LDH test was converted to TFD according to the yield criterion. Because a plane stress condition is assumed during sheet forming, an equation that can convert *ε_eq_* to *η* can be suggested even if only *ε*_1_ and *ε*_2_ are known.

(3) As ZIRLO™ is a highly anisotropic material, the anisotropy must be considered during the TFD conversion. Considering the anisotropy, the *η* value increases, and the TFD shifts to the right compared to the case where isotropy is assumed, which increases when the specimen width increases. In addition, in the case of the right part of the FLD (i.e., *ε*_1_ and *ε*_2_ > 0), the fracture strain also increases when anisotropy is considered.

(4) In order to confirm the effectiveness of the TFD converted to the equation, the TFD was obtained by fracturing four specimens of different shapes. As the influence of the friction coefficient cannot be ruled out in the FLD owing to the characteristics of the experiment, the effect of the friction coefficient on the forming limit was eliminated by stretching a characteristic specimen that exhibited a specific stress state. The isotropy assumption predicts an excessively low formability, while the converted TFD considering anisotropy is in close agreement with the TFD obtained from the characteristic specimens. This study can be expanded to help evaluate the formability of various stress states in production applications using HCP materials with high anisotropy such as Ti, Zr, Mg, and Be alloys. Furthermore, it is possible to simplify or replace the conventional TFD acquisition method, which was not previously standardized due to the specimen shape not specified, with a relatively well standardized FLD specimen.
